# K-Ras Peptide Mimotope Induces Antigen Specific Th1 and B-Cell Immune Responses against G12A-Mutated K-Ras Antigen in Balb/c Mice

**DOI:** 10.3390/vaccines9030195

**Published:** 2021-02-26

**Authors:** Pui Yan Siak, Kuan Yee Wong, Adelene Ai-Lian Song, Raha Abdul Rahim, Lionel Lian Aun In

**Affiliations:** 1 Department of Biotechnology, Faculty of Applied Sciences, UCSI University, Kuala Lumpur 56000, Malaysia; spysiak@hotmail.my (P.Y.S.); kuanyee96@gmail.com (K.Y.W.); 2 Department of Microbiology, Faculty of Biotechnology and Biomolecular Sciences, University Putra Malaysia, Selangor 43400, Malaysia; adelene@upm.edu.my; 3 Technical University of Malaysia Malacca, Malacca 76100, Malaysia; raha@upm.edu.my

**Keywords:** *KRAS*, *Lactococcus lactis*, tetanus toxoid, peptide-based vaccine

## Abstract

*KRAS* G12A somatic point mutation in adenocarcinomas is categorized clinically as ineligibility criteria for anti-epidermal growth factor receptor (EGFR) monoclonal antibody therapies. In this study, a modified G12A-K-ras epitope (139A) with sequence-specific modifications to improve immunogenicity was developed as a potential vaccine against G12A-mutant *KRAS* cancers. Additionally, coupling of the 139A epitope with a tetanus toxoid (TTD) universal T-cell epitope to improve antigenicity was also reported. To facilitate convenient oral administration, *Lactococcus lactis*, which possesses innate immunomodulatory properties, was chosen as a live gastrointestinal delivery vehicle. Recombinant *L. lactis* strains secreting a G12A mutated K-ras control and 139A with and without TTD fusion were generated for comparative immunogenicity assessment. BALB/c mice were immunized orally, and high survivability of *L. lactis* passage through the gastrointestinal tract was observed. Elevations in B-cell count with a concomitant titre of antigen-specific IgG and interferon-γ secreting T-cells were observed in the 139A treated mice group. Interestingly, an even higher antigen-specific IgA response and interferon-γ secreting T-cell counts were observed in 139A-TTD mice group upon re-stimulation with the G12A mutated K-ras antigen. Collectively, these results indicated that an antigen-specific immune response was successfully stimulated by 139A-TTD vaccine, and a TTD fusion was successful in further enhancing the immune responses.

## 1. Introduction

Kirsten Rat Sarcoma (*KRAS*) is one of the most frequently mutated human proto-oncogenes, and is highly prevalent in pancreatic, colorectal (CRC), prostate and non-small-cell lung carcinoma (NSCLC) [1]. This aberration predominantly occurs in codons 12 and 13 of the *KRAS* gene, specifically with a G12A, G12V, G12C, G12D, or G13D substitution in CRC. *KRAS* belongs to the RAS family, which encodes for a GTP-binding protein. It functions as a reversible plasma membrane-localized molecular switch that controls several downstream effector pathways, such as the Ras-Raf-MEK-ERK, mTOR, and PI3K/AKT pathways, thus affecting cell differentiation, proliferation, arrest, and apoptosis [1,2]. K-ras protein is activated when a GTP binds into its active region, forming an active KRAS-GTP complex. The switch is inactivated upon hydrolysis of GTP to GDP by GEFs/GRFs for the regulation of normal cell signalling. Oncogenic mutations in K-ras typically alters the GTP binding region, resulting in the inability to hydrolyze GTP, hence locking K-ras in a perpetually active state.

Due to the diversity of mutant KRAS variants and the current unavailability of effective direct inhibitors towards it, alternative treatment options for patients within this cohort is imperative. While anti-EGFR-targeted monoclonal antibodies, such as panitumumab and cetuximab, have found reasonable ground against cancers with an abnormal EGFR activation upstream of a wild-type *KRAS* (wt*KRAS*), cancer patients with a mutant *KRAS* showed no therapeutic benefit from such therapies, rendering mutant *KRAS* a predictive biomarker for negative therapeutic response in anti-EGFR therapy [3,4,5,6].

The development of immunotherapeutic vaccines targeting MHC-restricted tumor neoantigens such as mutant K-ras, which can potentially guide the immune system to recognize, attack, and develop memory based immune responses is greatly anticipated. In recent years, the use of native tumor antigenic epitopes derived from tumor associated antigens (TAAs) or tumor specific antigen (TSAs) was reported to successfully induce effective cytotoxic T-cell (CTL) responses, but no therapeutic benefit was observed in clinical trials [7,8,9,10,11]. This was mainly attributed to thymic selection and immunosuppression of TAAs or TSAs as self-antigens, and through tumor immune editing mechanisms.

Mimotopes, which are sequence modified mimics of natural tumor antigen epitopes can potentially improve antigenicity and overcome the immunosuppression problem. Such mimotopes can be designed with higher MHC binding affinity, thereby stimulating stronger cytokine responses and enhanced T-cells responses [12,13]. An ideal length of a mimotope vaccine is typically 8–20 amino acids, which is favorable for MHC-II binding and eases production [14]. To date, numerous peptide mimotopes have been developed against different antigen types, including EGFR, KRAS, HER2, CEA, PSA, MG7-Ag, and many others with improved MHC-binding affinity and presentation by antigen presentation cells (APCs) [15,16,17,18,19]. Furthermore, several studies have reported that poor immunogenicity issues affiliated with short peptide mimotope vaccines could be overcome through conjugation of established bacterial carrier molecules such as tetanus toxoid (TTD) and diphtheria toxoid (DTD) that contain universal T-cells epitopes, thus enhancing both humoral and cell-mediated responses [20,21].

The use of the lactic acid bacteria (LAB) as an oral vaccine delivery vehicle is a relatively new approach for vaccine delivery that has shown great promise. *Lactococcus lactis* is one of the LAB that carries a generally recognized as safe (GRAS) status approved by U.S. Food and Drug Administration (FDA) [22], and its use has been extended from the dairy industry to biomedical applications over the past decade. Among the advantages of using *L. lactis* as an expression and delivery system is its ability to survive passage through the harsh conditions of the gastrointestinal (GI) tract, its non-colonising properties, presence of only one housekeeping extracellular protease (*hrtA*) and absence of inclusion bodies [23]. In addition, the presence of a well-established *L. lactis* secretory system using Usp45 signal peptide (SP) also facilitates efficient extracellular secretion of heterologous proteins. This allows better interaction of the antigen with the target to induce stronger immune responses. *L. lactis* is also known to elicit innate immunomodulatory properties which confer an adjuvant effect, capable of inducing both mucosal and systemic immunity in gut-associated lymphoid tissue (GALT) [24,25], which positively activates anti-cancer mechanisms [26,27,28].

In the present study, the construction of recombinant *L. lactis* secreting a G12A-K-ras mutated peptide mimotope (termed 139A) vaccine candidate, its in vivo immunological assessment, and its survivability through the GI tract were reported. Furthermore, comparative immunological responses triggered by *L. lactis*:139A-K-ras mimotope with and without a TTD fusion were also evaluated.

## 2. Materials and Methods

### 2.1. Bacteria Strains, Plasmids and Growth Conditions

*L. lactis* NZ9000 obtained from University Putra Malaysia was cultured anaerobically at 30 °C in M17 broth (Merck, Darmstadt, Germany) containing 0.5% (*w*/*v*) glucose (Chemiz, Sham Alam, Malaysia) (GM17) [29]. For recombinant strains, 10.0 μg/mL of chloramphenicol (Cm) (Merck, Darmstadt, Germany) was supplemented [30]. XL 10-Gold *E. coli* (Stratagene, San Diego, CA, USA) was cultured aerobically at 37 °C in Luria-Bertani (LB) broth (HiMedia Laboratories, Mumbai, India), and for recombinant strains, 100.0 mg/mL ampicillin (Nacalai Tesque, Kyoto, Japan) was added. Details of plasmids and bacteria strains used and constructed in this study are listed in Table S1.

### 2.2. Cloning and Expression of Recombinant L. lactis

Primers used in this study are listed in Table S2. Oligonucleotide 139-A (YKLVVVPAAGVGKSA) which was designed previously [19] and TTD [20] were synthesised separately in pIDT:SMART vectors (Integrated DNA Technologies, Clareville, IA, USA). Recombinant pIDT:SMART plasmid was transformed via heat-shock into the X-10 GOLD *E. coli*. The cDNA of wt*KRAS* which was obtained from a previous study [31] was used as the template for PCR-based site direct mutagenesis to generate the first 50 codon of *KRAS* sequence with G12A mutation, incorporating restriction sites at both ends, and a 6× histidine tag at the C-terminal. PCR amplification was carried out using 0.2–1.0 μg template and the PCR mix contained 2.0 mM MgCl_2_, 0.2 mM dNTP mix, 0.05 mM primers and 0.02 U/μL Q5 Hi-Fidelity DNA polymerase (New England Biolabs, Boston, MA, USA). The resulting PCR product was then inserted into the pNZ8048 lactoccocal vector and cloned in *L. lactis* NZ9000 via electroporation at 2300 V with a time constant of 5 milliseconds [32]. Usp45, G12A, 139A and TTD were amplified from pNZ-Usp45-X, pNZ-G12A, pIDT:SMART-139A and pIDT:SMART-139A-TTD respectively. A 1:1 equimolar ratio of PCR products was subsequently used as a template for splicing overlap extension PCR to generate the fused fragment, Usp45-G12A and 139A-TTD. The amplified 139A and 139A-TTD were inserted separately into the pNZ-Usp45 resulting in pNZ-Usp45-139A and pNZ-Usp45-139A-TTD, while Usp45-G12A was inserted into pNZ8048, resulting in pNZ-Usp45-G12A. Positive transformants were confirmed by colony PCR and sequencing using pNZ8048 vector-specific primers. Recombinant *L. lactis* strains were cultured overnight at 30 °C in GM17 broth supplemented with 10.0 μg/mL Cm. The overnight grown recombinant *L. lactis* strains were subcultured into fresh GM17 media containing 10.0 μg/mL Cm at 1:10 ratio and incubated at 30 °C until log phase where the optical density at 600 nm (OD_600_) reached 0.4–0.5. Cultures were then induced with 40.0 ng/mL nisin for 6 h at 30 °C.

### 2.3. Protein Extraction and Western Blotting

Induced cultures were harvested by centrifugation at 3500× *g* for 10 min. Supernatant and cell pellet fractions were processed separately. The pellet was washed with ice-cold 1× phosphate buffered saline (PBS) and sonicated using the Q500 Sonicator (Qsonica, LLC, Newton Connecticut, CT, USA) for a total pulse time of 10–30 min with an interval of 10 s pulse on and 30 s pulse off cycle. Meanwhile, extracellular proteins were trichloroacetic acid (TCA) precipitated from the induced pellet free supernatant. One quarter (*v*/*v*) of 100% ice-cold TCA was added into the supernatant fraction and incubated at 4 °C for 1 h, followed by washing twice with one quarter (*v*/*v*) of ice-cold acetone. The washed precipitated protein was air-dried and resuspended in 1× PBS. Both intracellular and extracellular protein suspensions were mixed with sample buffer (Laemmli buffer and β-mercaptoethanol) and boiled at 95 °C, followed by separation on a 15% (*w*/*v*) tricine gel at 100 V for 5 h. Western blot was conducted by wet transfer of proteins onto PVDF membrane (Bio-rad, Hercules, CA, USA), blocked with 1% BSA and detected using KPL HisDetectorTM Nickel-HRP conjugate (1/10,000) (KPL, Milford, CT, USA) and TMB substrate (KPL, Milford, CT, USA). Secretion efficiency was determined by densitometry analysis of western blot results using Image J software.

### 2.4. Ni-NTA-HRP ELISA Specific Quantification of K-Ras Mimotope

Protein yields from recombinant *L. lactis* strains at the optimum hour were determined using the Ni-NTA-HRP enzyme-linked immunosorbent assay (ELISA). ELISA was conducted using HisDetector^TM^ Western Blot HRP Colorimetric Kit (KPL, Milford, CT, USA) according to the manufacturer’s instructions. A 96-well MaxiBinding microtiter plate (SPL Life Sciences, Pocheon, Gyeonggi, Korea) was coated with 100.0 μL of protein standards, intra and extra-cellular total protein samples (100.0 μg/mL) in separate wells and incubated for 1 h. After that, the plate was blocked with 200.0 μL of 1% (*w*/*v*) BSA for 30 min, followed by incubation with 200.0 μL of 2% (*w*/*v*) sucrose for 5 min. Liquid from wells were removed and air-dried for 2 h. His-tagged proteins were detected by incubation with 1:1000 diluted KPL HisDetector^TM^ Nickel-HRP in 1% (*w*/*v*) BSA for 30 min. Plates were then developed for 30 min with 100.0 μL of TMB substrate (Elabscience, Houston, TX, USA) and the reaction was stopped by adding 100.0 μL of 1N sulphuric acid. After that, absorbance readings were measured at 450 nm using FLUOstar^®^ Omega (BMG LABTECH GmbH, Ortenberg, Germany). K-ras mimotope concentrations of each well were calculated by interpolating sample values from the standard curve.

### 2.5. Mice Strains

A total of 20 five weeks old female Balb/c mice (body weight range of 19.0–21.0 g) were obtained from Animal Resource Unit, Faculty of Medicine, University Putra Malaysia and housed in Laboratory of Vaccines and Immunotherapeutics (LIVES) at Comparative Medicine and Technology Unit (COMeT), University Putra Malaysia. Husbandry and care of animals were performed in accordance to ARRIVE guidelines under Institutional Animal Care and Use Committee ethics approval and Animal Welfare Act 2015 (Act 772), Malaysia. All mice were housed in cages with corn cob bedding, and were provided with food and water *ad libitum*. All mice were acclimatized for 2 weeks prior to immunization. All experimental procedures were approved by Animal Ethical and Experimental Committee of University Putra Malaysia (2017-200309/UCSI/R/LILA).

### 2.6. Oral Immunization

Five groups (*n* = 4) of BALB/c mice were orally administered with nisin-induced recombinant live *L. lactis*:pNZ-Usp45-139A, *L. lactis*:pNZ-Usp45-G12A, *L. lactis*:pNZ-Usp45-139A-TTD, *L. lactis*:pNZ8048 and 0.2 M NaHCO_3_ pH 7.5 respectively. *L. lactis*:Usp45-139A and *L. lactis*:pNZ-Usp45-139A-TTD groups served as therapeutic test groups, whereas *L. lactis*:pNZ-Usp45-G12A served as the mutant *KRAS* control group. *L. lactis*:pNZ8048 and NaHCO_3_ groups served as the empty vector and negative control respectively. During each immunization, 100.0 μL of 2 × 10^11^ colony forming units (cfu) of each recombinant construct was delivered via oral gavage. Each mice group received 10 treatments in total over four weeks as depicted in Figure S1.

### 2.7. Detection of Recombinant L. lactis from Mice Feces

The mice group that was orally administered with *L. lactis*:pNZ-Usp45-G12A was used for in vivo examination of GI passage survivability. Fresh fecal pellets were collected from 4 mice before (0 h) and 3, 6, 9, 12, and 24 h from the first oral immunization, and 24 h after the second and third administration (Figure S1). Each feces sample was collected in a microcentrifuge tube, weighed, and dissolved in 1× PBS pH 7.5 (100.0 mg:1.0 mL). Feces samples were then homogenized by grinding with a glass rod and vortexing. Serial dilutions 10^−1^ to 10^−11^ were made using 1× PBS pH 7.5 and plated onto GM17 agar with 10.0 μg/mL Cm in triplicates and incubated at 30 °C for 2 days. CFU counts were measured and recombinant *L. lactis*:pNZ-Usp45-G12A strains were confirmed by colony PCR using vector-specific primers. Data were reported as log_10_ mean ± standard deviation of four biological replicates.

### 2.8. Isolation of Blood, Sera, Intestinal Wash Samples and Splenocytes

Mice were anaesthetized with a ketamine-xylazine cocktail before sample collection. Whole blood was collected from anaesthetized mice prior to immunization (Day 0) by retro-orbital puncture and post-euthanization (3 days after the last booster) by cardiac puncture. Following cervical dislocation, both lower GI tract and spleen were dissected out. GI wash samples were collected by flushing the GI tract with 2 mL ice-cold 1× PBS pH 7.5 with 1.0 M PMSF (Sigma-Aldrich, Merck, St. Louis, MO, USA) and centrifuged at 3000× *g* for 20 min at 4 °C. The spleen was washed with pre-chilled 1× PBS pH 7.5, followed by gentle dissociation through a cell strainer (Miltenyi Biotech, Sunnyvale, CA, USA). The cell strainer was washed 5 times with 1× PBS pH 7.5 to maximise recovery of splenocytes, centrifuged, and cultured in RPMI1640, 10% FBS supplemented with 100.0 units of penicillin and 100.0 μg/mL streptomycin. Cell viability was determined using a haemocytometer.

### 2.9. Immunophenotyping of T and B-Cell Populations

Lymphocytes in whole blood were surface stained with fluorochrome-conjugated antibodies according to the manufacturer’s instructions (BioLegends, San Diego, CA, USA). Mice whole blood (100.0 μL) was pre-treated with 1.0 μg of purified anti-mouse CD16/32 (BioLegend, San Diego, CA, USA) to block Fc-receptors, followed by cell surface staining with 0.125 μg of Brilliant Violet 421-conjugated anti-mouse CD3, 0.125 μg of APC/Fire 750-conjugated anti-mouse CD4, 0.25 μg of Brilliant Violet 510-conjugated anti-mouse CD8a, 0.0625 μg of APC-conjugated anti-mouse CD19, 0.15 μg of Brilliant Violet 605-conjugated anti-mouse CD25, 0.125 μg of PerCP/Cy5.5-conjugated anti-mouse CD335 and 0.5 μg of PE-conjugated anti-mouse FOXP3 antibodies (BioLegend, San Diego, CA, USA). After cell staining, red blood cells were lysed with 2.0 mL of RBC lysis buffer (BioLegend, San Diego, CA, USA), and stained cells were acquired using Novocyte^TM^ flow cytometer (ACEA Biosciences, San Diego, CA, USA). The data were analyzed using NovoExpress^®^ software version 1.3.0 (ACEA Biosciences, San Diego, CA, USA). Data were expressed as mean percentage ± standard deviation of three biological replicates, and Student’s *t*-tests were performed with a *p*-value ≤0.05 (*) representing statistically significant differences.

### 2.10. Detection of K-Ras-Specific Serum IgG and Intestinal IgA

K-ras mimotope specific IgA and IgG levels from GI wash and serum samples were measured using indirect ELISA. The 96-well MaxiBinding microtiter plates were coated overnight with 100.0 μL of respective K-ras peptides (20 μg/mL) for G12A, 139A and 139A-TTD. IgG and IgA plates were blocked with 2% (*w*/*v*) skim milk and 2% (*w*/*v*) bovine serum albumin (BSA) (Sigma-Aldrich, St. Louis, MO, USA) respectively. Serum samples and GI wash samples were added into the respective plates and incubated for 1 h at RT. Bound IgG or IgA were detected using 1:10,000 diluted HRP-labelled goat anti-mouse IgG (Santa Cruz Biotechnology, Dallas, TX, USA) and 1:250 diluted HRP-labelled goat anti-mouse IgA (Invitrogen, Carlsbad, CA, USA), respectively. The plates were then developed with TMB substrate (Elabscience, elabscience Houston, TX, USA), and the reaction was stopped by adding 1 N sulphuric acid. Absorbance was measured at 450 nm using FLUOstar^®^ Omega (BMG LABTECH GmbH, Ortenberg, Germany). Non-coated wells contained sera or GI wash sample without antigen coating, while non-sample wells contained coated antigens without sample. IgG and IgA net concentrations were calculated by interpolating sample absorbance values from the standard curve and subtracting the concentration of non-sample and non-coated wells. The average of three biological replicates of IgA and Day 0 normalized IgG concentrations were calculated. Data were expressed as mean IgG or IgA concentration ± standard deviation. Student’s *t*-tests were performed to measure significant differences with a *p*-value ≤ 0.05 (*) threshold.

### 2.11. Ex Vivo Antigen Stimulation and Detection of IFN-γ Producing T-Cells

Previous isolated splenocytes were activated with G12A mutated K-ras peptides. Splenocytes treated with phytohaemagglutinin-M (Biowest, Nouvelle-Aquitaine, France) served as a positive control, whereas non-stimulated splenocytes served as a negative background control. Interferon-gamma (IFN-γ) producing T-cells were quantified using the ELISpot kit (BD^TM^ Bioscience, San Jose, CA, USA) according to manufacturer’s instructions. Pre-coated anti-mouse IFN-γ mAb plates were added with 100.0 μL of 1 × 10^6^ cells/mL of isolated splenocytes. Splenocytes were then stimulated with 20 μg/mL of K-ras mimotopes for 24 and 72 h at 37 °C with 5% CO_2_. The positive control well was stimulated with 20 μg/mL of phytohaemagglutinin-M, while the negative control well contained unstimulated splenocytes. All plates were incubated with 2 μg/mL of biotinylated anti–mouse IFN-γ antibody for 2 h, followed by 1 h with streptavidin-alkaline phosphatase. T-cells secreting IFN-γ were detected using AEC substrate, and the number of spots formed on each well was counted using Immunospot Analyser (CTL, Beaverton, OR, USA). Data were reported as mean number of spots ± standard deviation of two replicates. Student’s *t*-tests were also performed with a *p*-value ≤ 0.05 (*) representing statistically significant differences over non-stimulated controls [33].

### 2.12. Ethics Approval and Consent to Participate

All the animal care and husbandry were conducted according to the “Animal research: Reporting of in vivo experiments (ARRIVE)” guidelines. All procedures in this pre-clinical study were approved by the Institutional Animal Care and Use Committee (IACUC) under University Putra Malaysia (2017-200309/UCSI/R/LILA).

## 3. Results

### 3.1. Expression and Extracellular Secretion of K-Ras Peptide Mimotopes by L. lactis NZ9000

**Figure 1:**
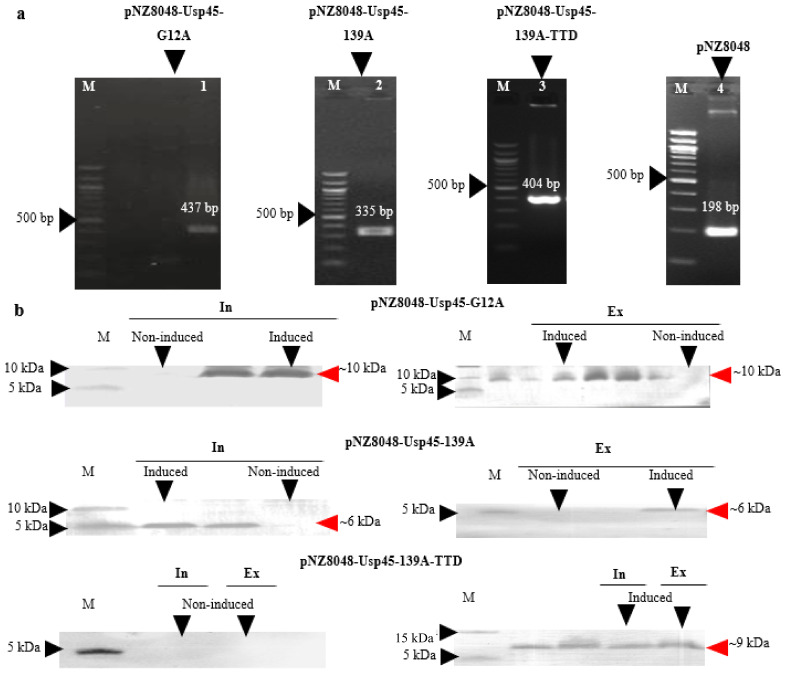
Recombinant *L. lactis* clones harbouring pNZ-Usp45-G12A, pNZ-Usp45-139A and pNZ-Usp45-139A-TTD. (**a**) Colony PCR of successful *L. lactis* transformants and pNZ8048 empty vector as negative control. (**b**) Western Blot of expressed Usp45-G12A, Usp45-139A and Usp45-139A-TTD in both intracellular and extracellular protein fractions detected via His-tag. Intracellular and extracellular band intensity ratios for Usp45-G12A, Usp45-139A and Usp45-139A-TTD can be found in Figure S2b. M: marker; In: intracellular protein fraction; Ex: extracellular protein fraction; Usp45: lactococcal extracellular secretion signal peptide; TTD: tetanus toxoid. Full membrane images can be found in Figure S3.

Usp45 SP was fused to the N-terminal of both control (G12A) and therapeutic (139A, 139A-TTD) K-ras mimotopes. Figure 1a shows the screening of positive *L. lactis* NZ9000 transformants by colony PCR using pNZ8048 vector-specific primers flanking the insert. The desired band size of positive transformants harbouring recombinant plasmids pNZ-Usp45-G12A, pNZ-Usp45-139A, and pNZ-Usp45-139A-TTD are 437 bp, 335 bp, and 404 bp respectively, while the 198 bp band represents an empty vector amplicon. No undesired mutations were detected following sequencing analysis (data not shown). Owing to the relatively low molecular weight of the desired peptide, tricine gels which offers higher resolution for small peptides were used for SDS-PAGE separation [34,35]. Positive transformants were then induced for desired protein expression which produced desired band sizes of 10 kDa, 6 kDa, and 9 kDa, corresponding to Usp45-G12A, Usp45-139A and Usp45-139A-TTD, respectively (Figure 1b). These results indicated that K-ras mimotopes were successfully expressed and secreted extracellularly under nisin induction. According to our previous study, expression of the desired protein increased with time and peaked at the 6 h mark [36]. Through Ni-NTA-HRP ELISA, a total of 28.12 μg/mL his-tagged K-ras mimotopes were detected after nisin induction for 6 h, of which 20.07 μg/mL of the K-ras mimotopes were found in the intracellular fraction, while 8.051 μg/mL were detected in the extracellular protein fraction (Ni-NTA-HRP ELISA graph can be found in Figure S2a). This translated to a secretion efficiency of approximately 28%.

### 3.2. Survivability of Recombinant L. lactis through the GI Tract

**Figure 2:**
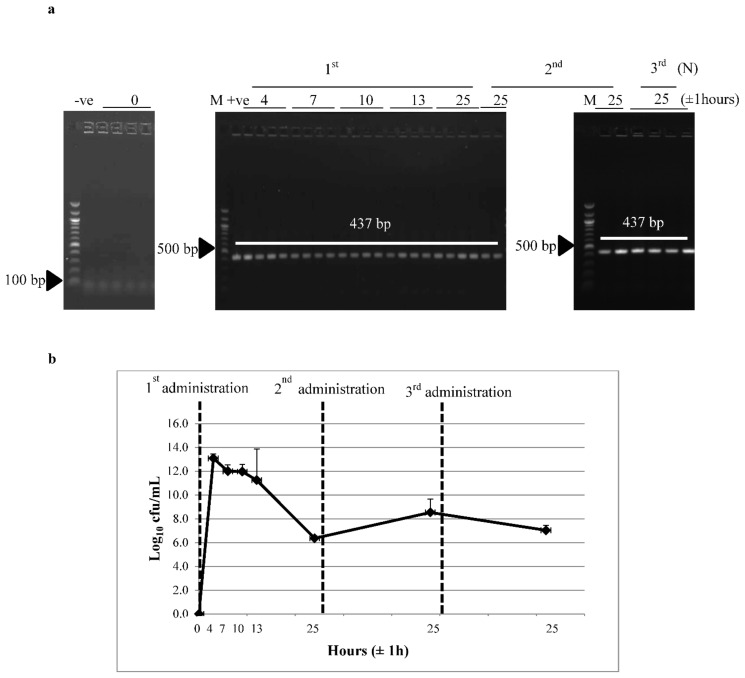
Recombinant *L. lactis* strain recovered from mice fecal samples before and across different time points after oral administration of *L. lactis*:pNZ-Usp45-G12A. (**a**) Colony PCR on recovered colonies from orally fed *L. lactis*:pNZ-Usp45-G12A mice faecal samples. (**b**) Viable counts of chloramphenicol-resistant recombinant *L. lactis* strain recovered from mice fecal samples across different hours after oral administration. Colony PCR was performed using pNZ8048 vector-specific primers. M: marker; –ve: L. lactis NZ9000; +ve: L. lactis:pNZ-Usp45-G12A. “N” represent the administration number. Results are expressed as log_10_ mean ± standard deviation of 4 mice.

Recombinant *L. lactis* harboring pNZ-Usp45-G12A was administered orally in mice and its survivability was monitored to confirm whether the administered recombinant *L. lactis* was able to reach the lower GI tract to trigger an immune response. Figure 2a depicts the agarose gel images of colony PCR using colonies isolated from mice fecal samples collected before and across different time points from first administration. The absence of creamy white colonies which is typical of lactococcal colonies and the desired PCR product indicated the absence of the recombinant vector in *L. lactis* before feeding. However, between 4 ± 1 to 25 ± 1 h after the first oral administration, white colonies were observed on Cm selective agar plates with a desired colony PCR band as the positive control (*L. lactis*:pNZ-Usp45-G12A) of approximately 437 bp (Figure 2a). Similarly, 25 ± 1 h after the second and third administrations, isolated white colonies were observed with the desired colony PCR bands. At 4 ± 1 h after administration, *L. lactis* (pNZ -Usp45-G12A) strain appeared at a maximum level of 10^13^ cfu/mL in feces (Figure 2b). At 7 ± 1 to 13 ± 1 h after the initial feeding, the strain appeared at 10^11^ cfu/mL and further decreased to 10^6^ to 10^9^ cfu/mL after 24 h. This indicated that 10^6^ to 10^9^ cfu/mL of *L. lactis* were recovered from mice feces after 24 h of each administration. These results also indicated that a high number of recombinant *L. lactis* was released 4 ± 1 h after administration and was further reduced after 6 h. Most of the bacteria were recovered within 25 ± 1 h. Recombinant *L. lactis* recovered from mice feces sample between 4 ± 1 and 25 ± 1 h after administration was at least 100-fold higher than the starting population (10^11^ cfu).

### 3.3. Oral Immunization of 139A-Secreting L. lactis Induces Elevation of B-Cell Population

**Figure 3:**
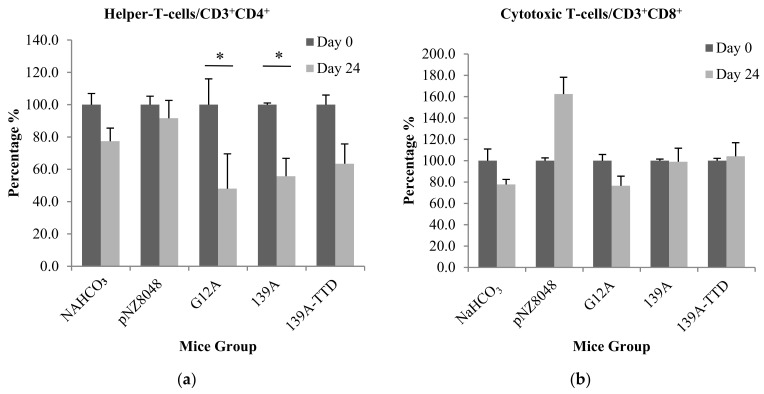

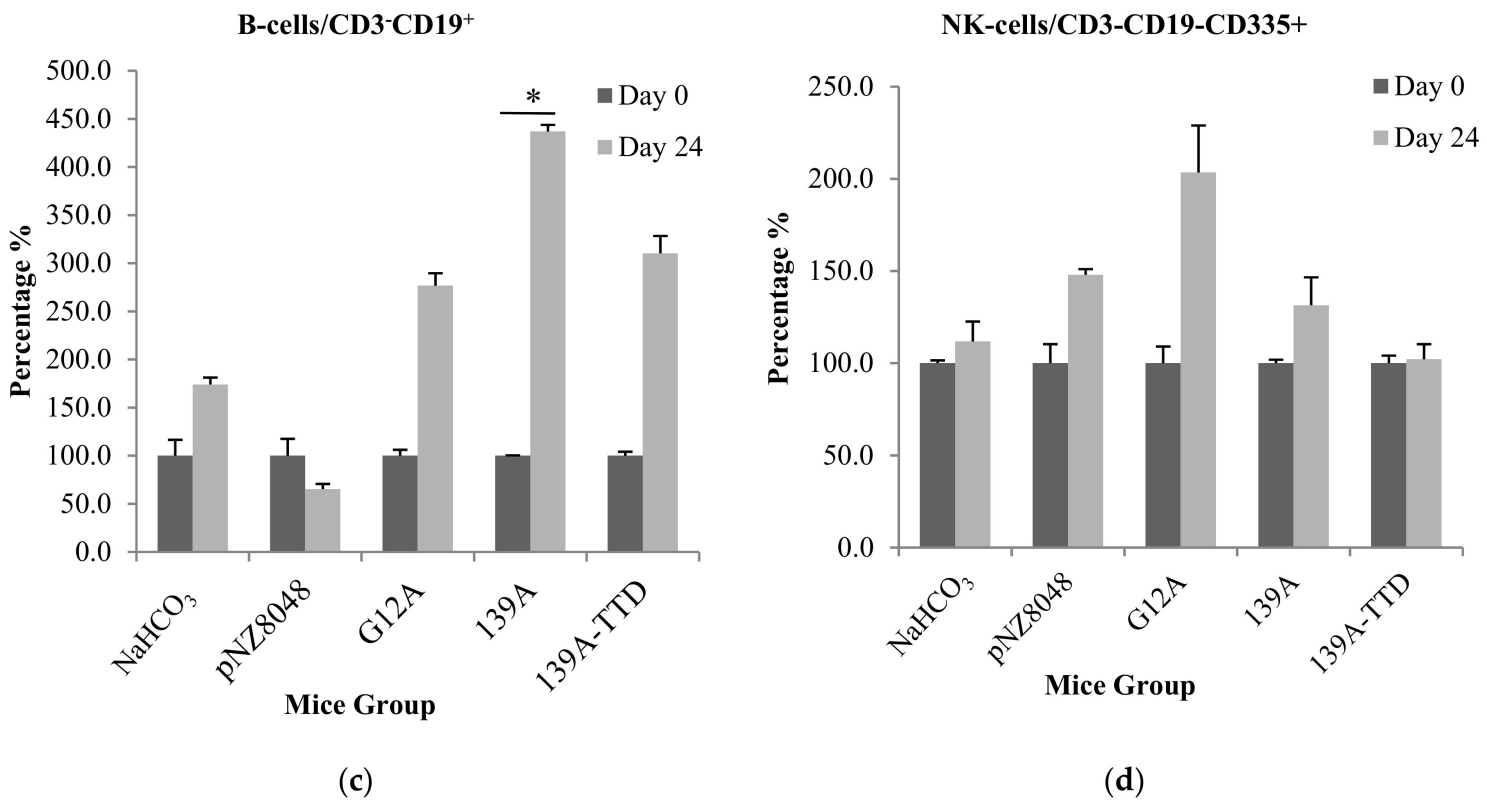
Immunophenotyping profile of mice whole blood before (pre-immunized) and after oral administration (post-euthanized) of *L. lactis* expressing K-ras control (G12A) and K-ras therapeutic mimotopes (139A, 139A-TTD). Bar charts depicts immune cell population of (**a**) B-cells/CD3^−^CD19^+^ (**b**) helper T-cells/CD3^+^CD4^+^ (**c**) cytotoxic T lymphocytes/CD3^+^CD8^+^ and (**d**) Natural killer cells/CD3^−^CD19^−^CD335^+^. Mice groups fed with sodium bicabornate solution and *L. lactis* harboring empty vector pNZ8048 served as negative controls. Results were expressed as mean percentage (%) ± standard deviation of immune cell populations of 3 mice between pre-immunized and post-euthanized mice whole blood. (*) represents significantly different mean values (*p*-value ≤ 0.05) between pre-immunized and post-euthanized mice whole blood.

Compared to pre-immunized blood samples, immunization of recombinant *L. lactis* expressing and secreting K-ras mimotopes was unable to elevate significant CD3^+^CD4^+^ cell populations in all mice groups as shown in Figure 3a. There was a significant decrease of CD3^+^CD4^+^ cell population in G12A and 139A mice groups, which may indicate suppression of helper T-cells. However, this supressed response was not significant when compared to negative control groups (NaHCO_3_ and recombinant *L. lactis* expressing empty vector pNZ8048) (Figure 3a). This indicated that the population of helper T-cells remained constant even after being immunized. Similarly, there was no significant change in CD3^+^CD8^+^ cell populations compared to pre-immunized (Day 0) whole blood as shown in Figure 3b. In Figure 3c, CD3^−^CD19^+^ cells populations were significantly elevated in the 139A immunized group, while no significant elevation was observed in negative control (NaHCO_3_), 139A-TTD and G12A groups. From Figure 3d, insignificant elevations of CD3^−^CD19^−^CD335^+^ (NK cell) population was observed in all mice groups. This could be attributed to the timing of sampling in relation to immune response kinetics. According to a previous study, NK cells are generally activated and transformed into effector cells 1.5 days after exposure to an antigen [37]. Therefore, minute elevation of NK cells (Figure 3d) could probably be elicited by the vaccine delivery host *L. lactis*. This is further supported by studies reporting that ingestion of probiotics such as *L. lactis* could stimulate NK cell production which is important in early lytic defence against foreign microbial, infected or malignant T-cells [31,38,39,40,41]. Collectively, significant elevations in B-cell population suggests the potential of 139A-K-ras mimotopes in triggering a humoral immune response. However, it is important to note that immunophenotyping assays only quantifies lymphocyte populations and cannot be used in standalone to represent antigen-specific activation. Therefore, the antigen-specific activation of existing lymphocyte populations, such as Th1 and B-cells, can only be detected by evaluating antigen-specific IFN-γ-secreting T-cells and antibody production levels. It was also important to note that administration of these vaccines did not confer any undesirable side effects and significant weight loss when comparing between groups. Details of mice weight changes pre- and post-immunization can be found in Figure S4.

### 3.4. Recombinant L. lactis Secreting 139A-TTD Enhances G12A-Mutant K-Ras-Specific Humoral Response in BALB/c Mice

**Figure 4:**
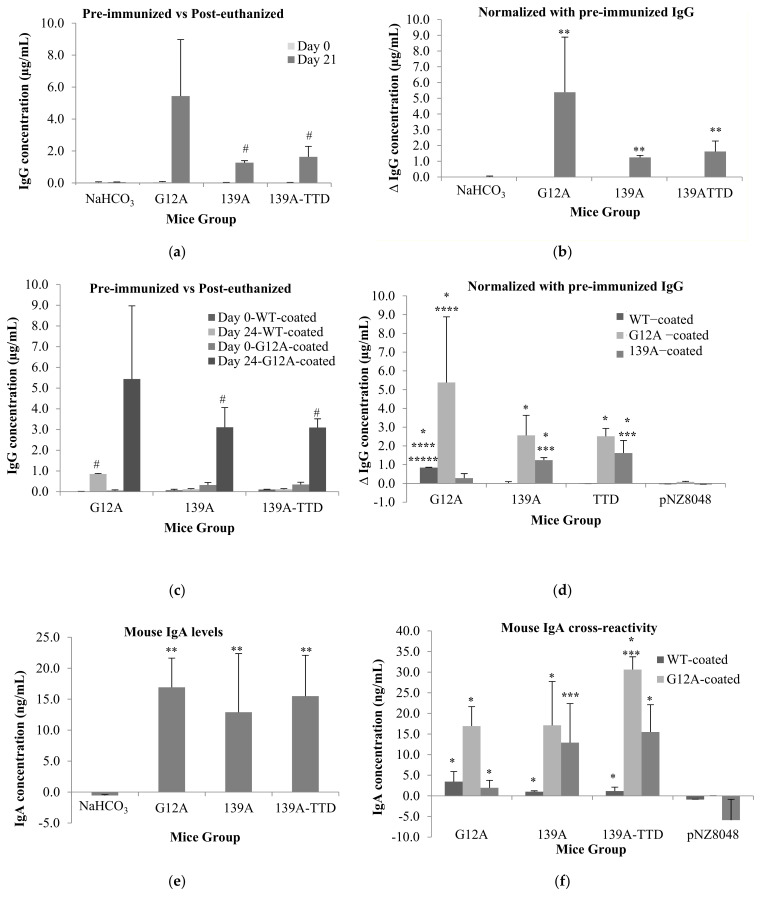
Levels of K-ras specific-serum IgG and intestinal IgA from mice immunized with recombinant *L. lactis* expressing K-ras control (G12A) and K-ras mimotopes (139A, 139A-TTD). Negative control groups: NaHCO_3_ and *L. lactis* NZ9000 harboring empty vector pNZ8048. Bar charts depicts (**a**) pre-immunized versus post euthanized IgG levels, (**b**) Day 0 normalized (Δ) IgG level, (**c**) pre-immunized versus post euthanized IgG cross-reactivity, (**d**) Day 0 normalized (Δ) IgG cross–reactivity, (**e**) IgA levels and (**f**) IgA cross–reactivity for all mice groups. Mice sera and GI wash samples were examined via indirect ELISA using respective K-ras peptides as coating antigens. Goat-anti-mouse IgG-HRP antibody was used as the secondary antibody. The absorbance was measured at 450 nm. Bars represent the mean ± standard deviation of 3 replicates. Mean values significant higher (*p*-value ≤ 0.05) from * pNZ8048, ** NAHCO_3_ and Day 0 group, *** G12A, **** 139A, ***** 139A-TTD, # pre-immunized group.

Sera and intestines of mice orally immunized with live recombinant *L. lactis* were tested for antigen-specific antibodies (IgG and IgA) via indirect ELISA using K-ras mutated peptides as coating antigens. Cross reactivity assessment was also conducted to determine whether specific antibodies elicited by immunized mice groups were able to target the native mutant G12A K-ras naturally encountered in tumor cells without attacking wtKRAS cells. In Figure 4a, antiserum IgG from 139A and 139A–TTD groups were significantly elevated when comparing between pre-immunization (day 0) and post-euthanization (day 24). This elevation (Figure 4a,b) was concomitant with elevations in B-cell population observed during immunophenotyping assays. When comparing mice groups at day 0 for normalized concentration of antiserum IgG against cross-reactivity assays (Figure 4d), G12A antiserum reacted the highest, followed by 139A-TTD and 139A groups. However, when comparing between pre-immunization (day 0) and post-euthanization (day 24) (Figure 4a,c), there were no significant difference in G12A-K-ras-specific IgG levels in G12A control group, while there was a significant (*p*-value ≤ 0.05) elevation of G12A-K-ras specific IgG in both 139A and 139A-TTD immunized groups (Figure 4c). For antigen-specific-IgA levels, G12A mice sera were shown to exhibit insignificantly higher IgA levels compared to 139A and 139A-TTD (Figure 4e). When observing the cross-reactivity results from Figure 4f, 139A antiserum cross reacted with the G12A-K-ras control peptide with a reactivity level comparable to the G12A antiserum. Interestingly, 139A-TTD antiserum displayed a significantly higher (*p*-value ≤ 0.05) reaction with the G12A-K-ras antigen compared to both G12A control and 139A groups. Cross-reactivity of both IgG and IgA antiserum from both 139A and 139A-TTD groups also indicated a negligible reaction against wt-K-ras, thus reassuring its specificity towards mutant G12A antigens. These results also confirm that a TTD fusion to 139A significantly enhances its G12A-specific IgA response.

### 3.5. Re-Stimulation of 139A-TTD Immunized Mice Splenocytes Induces G12A-Mutant K-Ras-Specific T-Cell Activation

**Figure 5:**
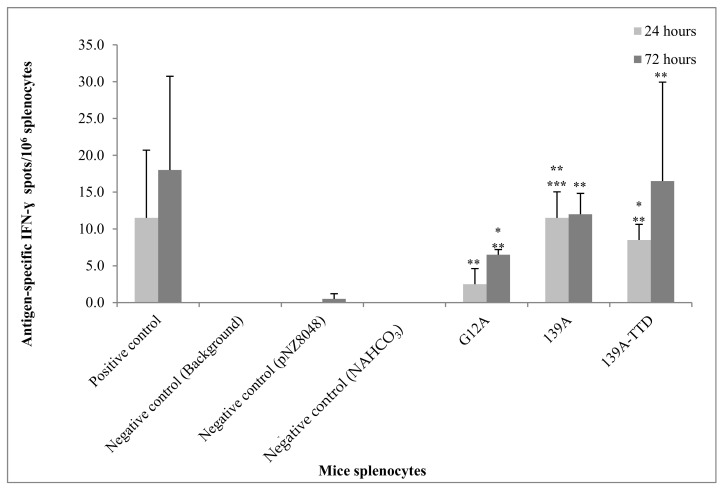
G12A-K-ras-specific cell-mediated immune (Th1) response of immunized mice. G12A-specific IFN-γ secreting T-cells were detected as spots via ELISpot assay. All immunised splenocytes were re-stimulated with G12A-K-ras control peptides. Positive control: splenocytes stimulated with PHA; negative control: (i) *L. lactis* expressing empty vector (pNZ8048) (ii) NAHCO_3_ immunized splenocytes (iii) medium (background) stimulated with G12A-K-ras control mimotopes. Data are presented as mean ± standard deviation of duplicate assays. No spots were observed in all non-stimulated splenocytes. Mean values significant higher (*p*-value ≤ 0.05) from * pNZ8048, ** NAHCO_3_, ** background control, ** non-stimulated and *** G12A group.

The assessment of antigen-specific IFN-γ secreting Th1 is important to demonstrate the potential use of recombinant *L. lactis* expressing 139A-K-ras mimotopes or it counterparts in triggering antigen-specific cell-mediated responses, including both Th1 and CTLs. Therefore, antigen induced lymphoproliferation and IFN-γ ELISpot assays were conducted. In order to assess antigen-specific T-cell activation, splenocyte cultures from immunized mice were re-stimulated with the same G12A-mutant K-ras antigen in vitro. In Figure 5, IFN-γ secreting T-cells were either significantly lower or undetectable in negative control immunized mice splenocytes (NaHCO_3_ and recombinant *L. lactis* harboring empty vector) upon antigen re-stimulation. Significantly higher (*p*-value ≤ 0.05) amounts of IFN-γ secreting T-cells were observed in 139A or 139A TTD groups compared to non-stimulated mice splenocytes. The number of IFN-γ secreting T-cells in 139A immunized mice splenocytes was also significantly greater (*p*-value ≤ 0.05) than G12A control immunized mice splenocytes after 24 h of re-stimulation. No significant difference was observed when comparing the number of IFN-γ secreting T-cells between 24 h and 72 h of stimulation. Therefore, ELISpot results indicated that G12A-specific IFN-γ secreting T-cells could be induced by both 139A and 139A-TTD vaccine candidates 24 h post-stimulation.

## 4. Discussion

The poor immunogenicity of cancer vaccines and immunotolerance towards self-antigens such as mutant K-ras has made them inefficient in triggering a strong immune response when administered without further sequence modifications. In order to overcome immunotolerance towards native mutant K-ras found on tumorigenic cells, a more immunogenic form of mutant K-ras epitope which mimics the natural mutant K-ras epitope was designed and optimized through in silico predictions [19]. This modified version, termed a mimotope, possesses an additional single amino acid substitution (G10P) flanking the native G12A mutation. It was then further packaged for delivery as an oral vaccine using food grade bacteria to potentially strengthen its immune response, thus addressing both concerns highlighted above.

Many studies have demonstrated the ability of *L. lactis* in delivering target proteins to the lower GI tract to trigger protective immunity [28,42,43]. The lower part of the GI tract contains microfold (M) cells which consist of a network of Peyer’s patches that are rich in APCs and T-cells [44,45]. Antigens secreted by the *L. lactis* or whole cells can be internalized by the mucosal surface barrier through uptake by M cells and reproduce in phagocytic cells, thereby triggering a dynamic immune response towards the antigen [46,47,48,49]. However, intracellular localized antigen-based vaccines generally generate a significantly lower immunization efficacy compared to extracellularly accessible antigens. The employment of secretory expression systems has been found to overcome this limitation by enabling larger scale extracellular delivery of antigens with minimal protein degradation, since only a single housekeeping extracellular protease is present in *L*. *lactis* [50]. To enable the secretion of K-ras peptide mimotope in *L. lactis*, the N-terminal SP Usp45, which directs the precursor to the secretion machinery was used [51,52]. Usp45 is one of the most widely used lactococcal SP owing to its relatively high heterologous protein secretion efficiency [29,51,52,53,54,55,56,57,58].

The survivability of *L. lactis* passage through the hostile condition in the body is an important characteristic of an oral live vaccine host to successfully deliver the expressed antigen to immune cells along the lower GI tract. In fact, most of the orally administered *L. lactis* will be greatly reduced when passing through the stomach and GI tract. The first physiological obstacle encountered is the low pH in the stomach, while the upper intestinal tract which contains bile salt and digestive enzymes will be the main barrier prior to the lower GI tract [59]. There are studies which reported that *L. lactis* has the capability of tolerating these stressful conditions and are comparable to other intestinal bacteria [60,61]. Another in vitro study which examined the survivability of recombinant *L. lactis* in simulated sequential gastric conditions for 2 h followed by 4 h of incubation under intestinal conditions, revealed that only 20% of the original amount of bacteria survived [62]. In the present study, recombinant *L. lactis* was evidenced to possess good GI resistance since high amounts of viable *L. lactis* were recovered in mice fecal samples 24 h after oral administration. In addition, recombinant *L. lactis* recovered from mice feces sample was found to be 100-fold higher than the starting population (10^11^ cfu). This demonstrated that recombinant *L. lactis* being fed were actively dividing, remained metabolically active and can be continuously expressed and secreted throughout the GI tract to trigger immune responses. In order to elicit an immune response, it was reported that at least 10^5^ cfu of metabolically viable bacteria must reach the lower GI tract [63,64]. Collectively, this suggested that *L. lactis* is a promising candidate for oral vaccine delivery.

In vivo assessment of the K-ras mimotope vaccine in triggering an active immune response was found to significantly activate a G12A-specific humoral response, as observed through increasing B-cell populations and a concomitant titre of G12A-specific IgG and IgA against the G12A-K-ras antigen. In addition, when vaccinated mice splenocytes were subjected to antigen re-stimulation, a significant elevation of G12A-K-Ras-specific IFN-γ-secreting T cell response was observed. These data demonstrated that immunization of 139A-K-ras mimotopes could enhance the stimulation of both antigen-specific IgG production and IFN-γ-secreting T-cells in mice against the native mutant G12A-K-ras antigen. These findings also suggested that the modification of G12A-K-ras with a G10P amino acid substitution flanking the mutation site could evidently enhance antigenicity as predicted in silico. Moreover, the fusion of TTD with modified G12A-K-ras (139A) further enhanced responses against the G12A-K-ras antigens. This proves the efficacy of a universal T cell epitope sequence of TTD as an immunogenicity enhancer against its conjugated antigen, where similar findings have been demonstrated [20].

The use of *L. lactis* as a vaccine delivery vehicle through the oral route was also able to induce intestinal mucosal immunity associated with a significant G12A-specific IgA production. These intestinal IgAs are protease resistant, thereby making them important as a first line of defense against pre-malignant tumor cells and lesions [65]. There are increasing number of studies reporting that tumor antigen identification for cancer vaccine development have shifted the paradigm of not only stimulating T-cell immunity for effective anti-tumor CTL response, but also to towards humoral immunity which is now proven to be important in tumor eradication [65,66,67,68].

In conclusion, the present in vivo immunogenicity assessment of the 139A-TTD vaccine candidate has supported the hypothesis that an active immune response can be stimulated following oral immunization of live recombinant *L. lactis*. Nevertheless, its anti-cancer prophylactic or therapeutic efficacy can only be validated following subsequent pre-clinical disease challenge studies. This study also reinforces the potential of *L. lactis* as a good vaccine carrier, thereby providing the basis for the exploratory development of other safe, inexpensive, and effective oral vaccines. Efforts are currently underway to (i) further expand the 139A-TTD vaccine into a multivalent tandem vaccine that covers other highly prevalent KRAS mutational variants, such as G12C, G12D, G12V, and G13D, (ii) evaluate the anti-cancer efficacy of the 139A-TTD vaccine in combination with checkpoint inhibitors, and (iii) utilize alternative lactococcal food grade vectors without an antibiotic resistance gene.

## Data Availability

The data presented in this study are available in this article and Supplementary Materials.

## Supplementary Materials

The following are available online at https://www.mdpi.com/2076-393X/9/3/195/s1, Table S1: Bacteria host strains and plasmids used or constructed in this study, Table S2: List of PCR oligonucleotide primers used in this study, Figure S1: Schedule of immunisation and samples collection including blood, feces, GI tract and spleen.

## Abbreviations

*Ad libitum*: As desired; APCs: Antigen presenting cells; APS: Ammonium persulfate; ARRIVE: Animal research: Reporting of in vivo experiments; BALB/c: Bagg albino strain with genotype c; bp: Base pair(s); Caco-2: Continuous line of heterogeneous human epithelial colorectal adenocarcinoma cells; CD: Cluster of differentiation; cfu: Colony forming unit; Cm: Chloramphenicol; Cm^r^: Chloramphenicol resistance; COMeT: Comparative Medicine and Technology Unit; CRC: Colorectal cancer; CTLs:Cytotoxic T lymphocytes; DNA: Deoxyribonucleic acid: dNTP: Deoxyribonucleotide triphosphate; EGF: Epidermal growth factor: EGFR: Epidermal growth factor receptor; ELISA: Enzyme-linked immunosorbent assay; ELISpot: Enzyme-linked immunosorbent spot assay; ERK: Extracellular signal-regulated kinase; et al.: et al.ia, which means “and others”; Fc: Immunoglobulin-like receptor; FDA-Food and Drug Administration; g: Gram; *g*: Relative centrifugal force; GM17: M17 agar or broth, supplemented with 0.5% (*w*/*v*) glucose; GALTs: Gut-associated lymphoid tissues; GAPs: GTPase activating proteins; GDP: Guanosine diphosphate; GEFs: Guanine nucleotide exchange factors; GI: Gastrointestinal; gPCR: Gradient PCR; GRAS: Generally recognised as safe; GRFs: Guanine nucleotide exchange factor; G-SGM17: M17 supplemented with sucrose, glucose and glycine; GTP: Guanosine triphosphate; GTPase: Guanosine triphosphatase; HLA: Human leukocyte antigen; HRP: Horseradish peroxidase; *hrtA: L. lactis housekeeping protease gene*; i.e.,: id est, which means “that is”. IACUC: Institutional Animal Care and Use Committee; IFN-γ: Interferon-gamma; IgA: Immunoglobulin A; IgG: Immunoglobulin G; IL: Interleukin; K-ras: Protein encoded from *KRAS* gene; K_2_EDTA: Dipotassium ethylenediaminetetraacetic acid; kb: Kilo base pair(s); kDa: kilo Dalton; KRAS: Kirsten rat sarcoma viral oncogene homolog; L: Litre; *L. lactis*: *Lactococcus lactis*; LAB: Lactic acid bacterium; LIVES: Laboratory of Vaccines and Immunotherapeutics; M: Molar; m: Metre; mAbs: Monoclonal antibodies; MAPK: Mitogen-activated protein kinase; mCRC: Metastatic colorectal cancer; MEK: Mitogen-activated protein kinase; MgCl_2_: Magnesium chloride; MHC: Major histocompatibility complex; mRNA: Messenger Ribonucleic acid; mt: Mutant; mt*KRAS*: Mutated *KRAS* gene found in cancer patient; mtK-ras: Mutated *KRAS* protein (antigen) found in cancer patient; N: Normality, also known as equivalent concentration; n: Sample size; NaHCO_3_: Sodium hydrogen carbonate; NICE: Nisin-controlled gene expression system; NK: Natural killer cells; NSCLC: Non-small cell lung carcinoma; NTA: Nitrilotriacetic acid; OD_600_: Optical density at wavelength of 600 nm; PBMC: Peripheral blood mononuclear cells; PBS: Phosphate-buffered saline; PCR: Polymerase chain reaction; PE: Phycoerythrin; PerCP/Cy5.5: Peridinin chlorphyll protein/Cy5.5, also known as TruRed; PI3K: Phosphoinositide 3-kinase or phosphatidylinositol-4,5-bisphosphate 3-kinase; PMSF: Phenylmethylsulfonyl fluoride; PSA: Prostate-specific antigen; PVDF: Polyvinylidene difluoride; Raf: Rapidly accelerated fibrosarcoma; RAS: Rat sarcoma; RBC: Red blood cell; RE: Restriction enzyme: RT: Room temperature; SDS-PAGE: Sodium dodecyl sulfate-polyacrylamide gel electrophoresis; SGM17: G-M17 broth supplemented with 0.5 M sucrose and 2% (*w*/*v*) glycine; SOE-PCR: Splicing by overlap extension-polymerase chain reaction; SP: Signal peptide; SPnuc: Nuc signal peptide; SPExp4/SPK1/SP310: Lactococcal signal peptide; SMD: Site direct mutagenesis; T-regs: Regulatory T-cells; TAAs: Tumor-associated antigens; *Taq*: DNA polymerase originally isolated from *Thermus aquaticus*; TCA: Trichloroacetic acid; Th: T helper; tK-ras: therapeutic K-ras mimotopes designed to target for mutated K-ras cancer; TMB: 3,3′,5,5′-Tetramethylbenzidine or 3,3′,5,5′-Tetramethyl[1,1′-biphenyl]-4,4′-diamine; TSAs: Tumor-specific antigens; TTD: Tetanus toxoid; TTFC: Tetanus toxin fragment C; U.K.: United Kingdom; U.S.: United States; Usp45: Ubiquitin specific peptidase 45; UV: Ultraviolet; V: Volts; *v*/*v*: Volume per volume; *w*/*v*: Weight per volume; wt: Wild-type; wt*KRAS*: Wild-type *KRAS*.
