# Supplementary material for: K-Ras Peptide Mimotope Induces Antigen Specific Th1 and B-Cell Immune Responses against G12A-Mutated K-Ras Antigen in Balb/c Mice
Source: Vaccines (Basel). 2021 Feb 26;9(3):195. doi: 10.3390/vaccines9030195 (PMC7996567; doi:10.3390/vaccines9030195)
Supplement: Supplementary file 1 [file vaccines-09-00195-s001.pdf]

Supplementary Table S1

Bacteria host strains and plasmids used and constructed in this study.

Host strains	Plasmids	Characteristics	Source
L. lactis NZ9000	-	*L. lactis* subsp. *cremoris* (derivative strain of MG1363, carrying *nisRK* genes on the chromosome).	University Putra Malaysia
L. lactis NZ9000	pNZ8048	*L. lactis* subsp. *cremoris* (derivative strain of MG1363, carrying *nisRK* genes on the chromosome).	University Putra Malaysia
L. lactis NZ9000	pNZ-Usp45-NucA	P_{nisA}/Cm^r pNZ8048 derivative containing Usp45-NucA	University Putra Malaysia
XL-10 GOLD *E. coli*	pIDT-SMART-139A mimotopes	$Amp^{R,}$ pIDT-SMART derivative containing synthesized t*K-Ras* mimotopes	Present study
XL-10 GOLD *E. coli*	pIDT-SMART-TTD	$Amp^{R,}$ pIDT-SMART derivative containing synthesized toxoid	Present study
L. lactis NZ9000	pNZ-wt*KRAS*	$P_{nisA}/Cm^{r,}$ pNZ8048 derivative containing wt*KRAS*	[37]
L. lactis NZ9000	pNZ-G12A	$P_{nisA}/Cm^{r,}$ pNZ8048 derivative containing fusion of Usp45 and natural mutant G12A-*KRAS* in exon 2	Present study
L. lactis NZ9000	pNZ-Usp45	P_{nisA}/Cm^r pNZ8048 derivative containing Restriction site-modified Usp45	[37]
L. lactis NZ9000	pNZ-Usp45-G12A	P_{nisA}/Cm^r, pNZ8048 derivative containing fusion of Usp45 and natural mutant G12A-*KRAS* in exon 2	Present study
L. lactis NZ9000	pNZ-Usp45-139A	P_{nisA}/Cm^r, pNZ8048 derivative containing fusion of Usp45 and 139A mimotopes	Present study
L. lactis NZ9000	pNZ-Usp45-139A-TTD	P_{nisA}/Cm^r, pNZ8048 derivative containing fusion of Usp45 and 139A mimotopes-tetanus toxoid	Present study

Supplementary Table S2

List of PCR oligonucleotide primers used in this study.

Name	Forward (5'-3')	Reverse(5'-3')	T_m (^oC)	Amplicon Size (bp)	Template
pNZ8048	TATTGTCGATAACGCGAGCAT	CGTTTCAAGCCTTGGTTTTC	59	Vary with insert size	Recombinant pNZ8048 plasmid
SDM	TGGGTACTGCAGAAATGACTGAATATAAACTTGTGGTAGTTGGAGCTGCTGCCGTAG	CTATATACTAGTCTAATGATGATGATGATGATGGGTTTCTCCATCAATTACTAC	57	180	wt*KRAS* cDNA
**For construction pNZ-Usp45- therapeutic *KRAS* (139A)**					
Usp45[a]	CACTCACCATGGGCATGAAAAAAAAG	TTATATTCGGCATGCAGCGTAAACAC	56	110	pNZ8048-Usp45-Xgene
Therapeutic *KRAS* mimotopes	GCCTCTGGGTAGCATGCGAATATAAATTAG	TAGAACTAGTCTAATGATGATGATGATGATGA	59	193	pIDTSMART-139-A
**For construction pNZ-Usp45-mt*KRAS* (control)**					
Usp45[b]	GCACTCACCATGGGCATGAAAAAAAAGATTATCTC	GTTTATATTCAGTCATGCATGCAGCGTAAACACC	56	118	pNZ8048-Usp45-Xgene
mt*KRAS* mimotopes	TTACGCTGCATGCATGACTAATATAAACTTGTGG	AGCTTGAGCTCTCTAGAACTAGTCTAATGATGATG	61	207	pNZ8048-G12A
SOE-Usp45-mt*KRAS*	GCACTCACCATGGGCATGAAAAAAAAGATTATCTC	AGCTTGAGCTCTCTAGAACTAGTCTAATGATGATG	59	299	Two overlapping PCR products
**For construction of pNZ-Usp45- therapeutic *KRAS*-TTD**					
Mimotopes-TTD-B	GCCTCTGGGTAGCATGCGAATATAAATTAG	AAAATTATTACTAGTATGATGATGATGATGATGAGC	67	98	pIDTSMART -139A
TTD	CATCATCATCATCATCATACTAGTAATAATTTTACAGTTTCATTTTGG	AAGCTTCTAATGATGATGATGATGATGTGTTTCTAAATGTGAAGCTGA	62	144	pIDTSMART-TTD
SOE-mimotopes-TTD	GCCTCTGGGTAGCATGCGAATATAAATTAG	AAGCTTCTAATGATGATGATGATGATGTGTTTCTAAATGTGAAGCTGA	66	179	Two overlapping PCR products

Supplementary Figure S1

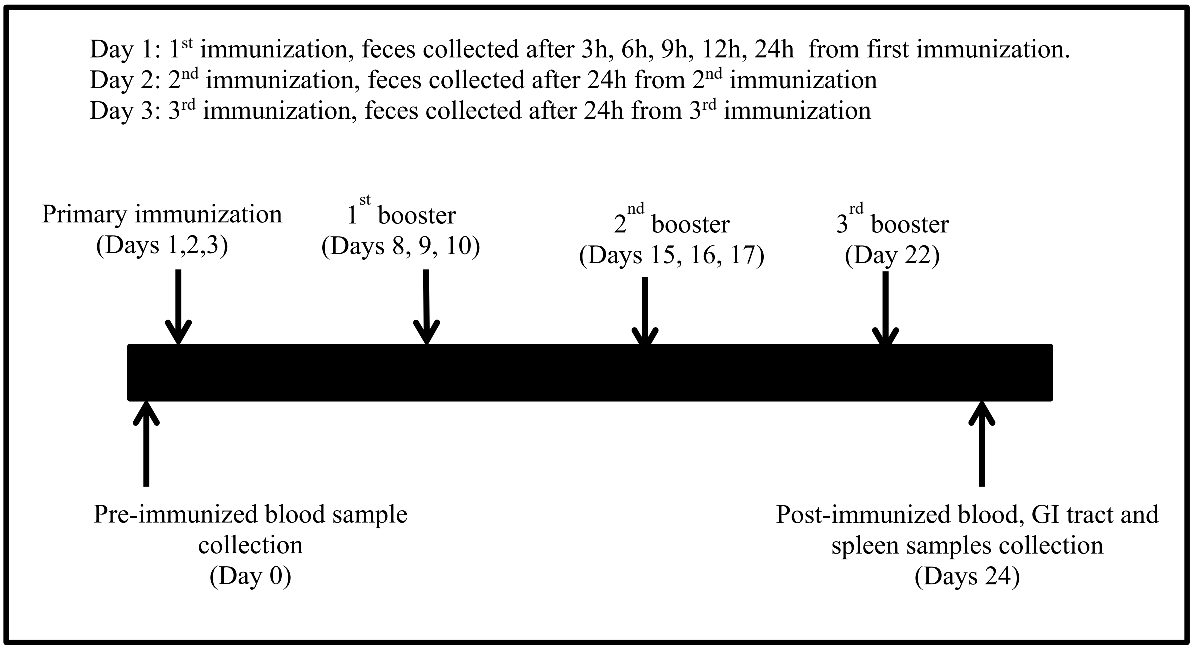

Schedule of immunization and sample collection including blood, feces, GI tract and spleen. To evaluate immune response raised by 139A-tK-ras mimotopes and its counterparts, 5 groups (n=4) of BALB/c mice were tested. Each group were separately fed with: $NaHCO_3$, recombinant *L. lactis* expressing control G12A-K-ras, 139A-tK-ras, 139A-tK-ras-TTD and empty vector pNZ8048. Each group was orally administered on day 1/2/3, and boosted on days 8/9/10, 15/16/17 and 22.

Supplementary Figure S2

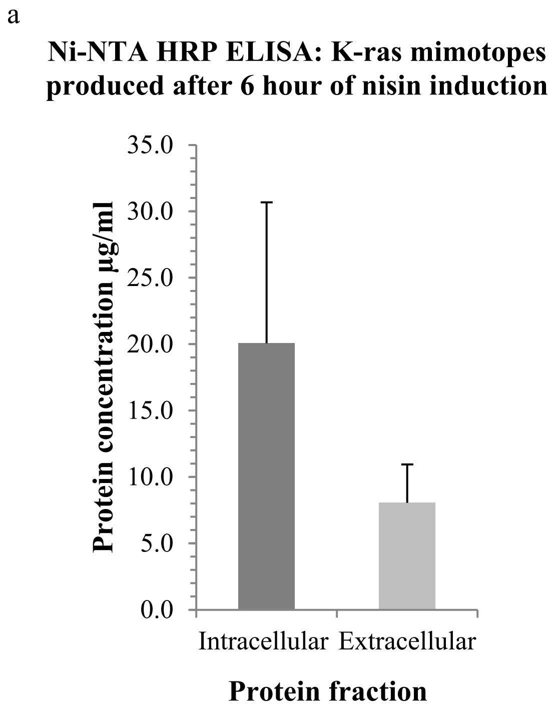

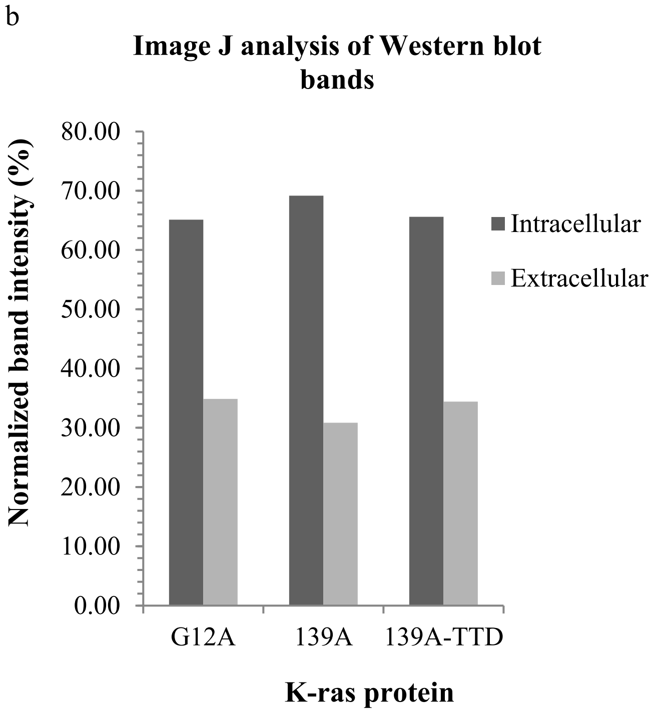

K-ras mimotopes produced after 6 hour of nisin induction. a) Concentration of K-ras mimotopes b) Image J analysis of Western blot bands representing intra- and extra-cellular K-ras mimotopes produced after 6 hour of nisin induction.

Supplementary Figure S3

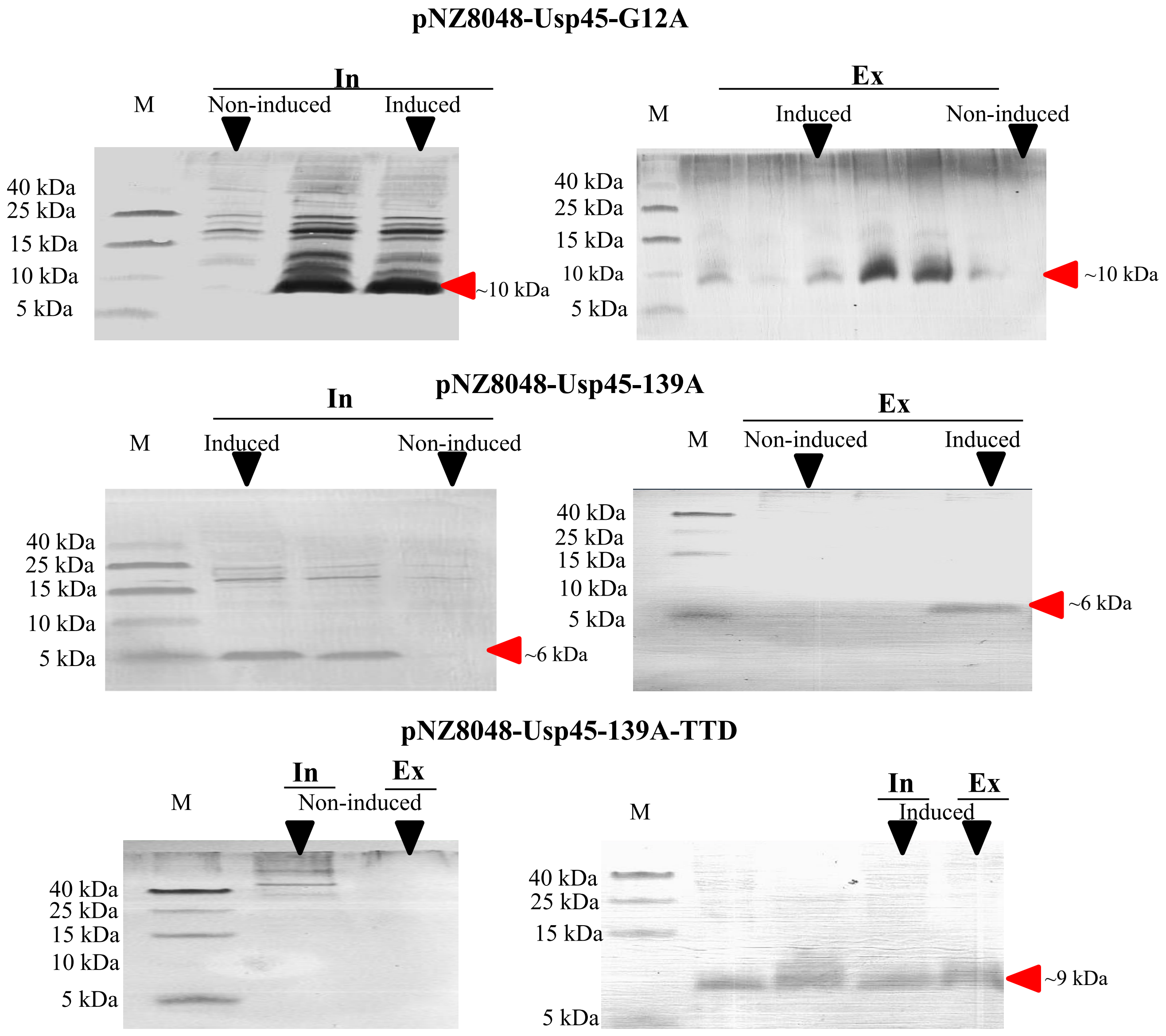

Recombinant *L. lactis* clones harbouring pNZ-Usp45-G12A, pNZ-Usp45-139A and pNZ-Usp45-139A-TTD. Western blot of expressed Usp45-G12A, Usp45-139A and Usp45-139A-TTD in both intracellular and extracellular protein fractions were detected via His-tag markers. M: Protein ladder; In: intracellular protein fraction; Ex: extracellular protein fraction; Usp45: lactococcal extracellular secretion signal peptide; TTD: tetanus toxoid.

Supplementary Figure S4

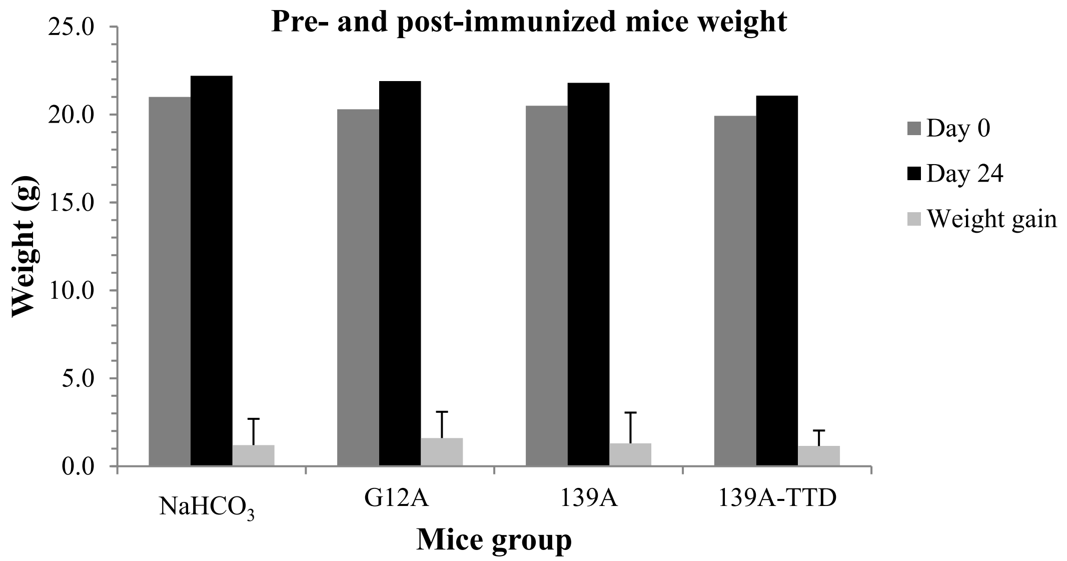

Average weight of 4 mice during pre- and post-immunized. No significance weight different between control and experimental groups.
